# HIV-1 RNA processing as therapeutic target: characterization of small molecule modulators of HIV-1 RNA processing and transport

**DOI:** 10.1186/1742-4690-10-S1-O45

**Published:** 2013-09-19

**Authors:** Raymond Wong, Ahalya Balachandran, Matthew Haaland, Peter Stoilov, Alan Cochrane

**Affiliations:** 1 Molecular Genetics, University of Toronto, Toronto, Ontario, Canada; 2 Biochemistry, West Virginia University, Morgantown, WV, USA; 3 Laboratory Medicine & Pathobiology, University of Toronto, Toronto, Ontario, Canada

## Background

Control of RNA processing plays a central role in the expression of the HIV-1 genome. From a single 9kb transcript, more than 40 mRNAs are generated through suboptimal splicing, a subset of which are dependent upon the HIV-1 factor Rev for export to the cytoplasm. To identify small molecule modulators of HIV-1 RNA processing, we performed a screen to isolate compounds capable of inhibiting HIV-1 gene expression post-integration.

## Materials and methods

Initial screens were performed in the HeLa rtTA HIVΔmls cell line containing a doxycycline inducible HIV-1 provirus. Effects of compounds on HIV-1 expression were analyzed using p24 ELISA, western blots, *in situ* hybridization and qRT-PCR. To confirm that the responses observed were not unique to this cell line, assays were repeated in a chronically infected CD4+T cell line (24STNLESG).

## Results

Two compounds displayed significant suppression of HIV-1 gene expression; 8-azaguanine, and 2-[2-(5-nitro-2-thienyl)vinyl]quinoline (5350150). Both compounds significantly reduced the production of HIV-1 Gag and Env with limited effects on Rev and Tat accumulation. While 8-azaguanine, was found to induce a significant alteration in the levels of unspliced, singly spliced and multiply spliced HIV RNAs, consistent with inducing an oversplicing phenotype, 5350150 had a more limited effect. Similar responses were seen using two distinct HIV-1 infected cell lines, suggesting that the response is conserved across multiple cell types. The lack of effect of both compounds on Rev and Tat levels while reducing expression of the HIV-1 structural proteins suggested that Rev function was being affected. Consistent with this hypothesis, both 8-azaguanine and 5350150 induced cytoplasmic accumulation of Rev suggesting an alteration in Rev’s ability to shuttle between the nucleus and cytoplasm and blocked nuclear export of HIV-1 genomic RNA as measured by *in situ* hybridization. Parallel analyses did not detect changes in the localization of known shuttling host proteins (hnRNP A1, SRp20) and only limited alterations of host RNA alternative splicing, suggesting that HIV-1 processing is highly sensitive to the effects induced by these compounds. To validate the anti-viral activity of these compounds, their capacity to suppress replication of HIV-1 isolates in PBMCs is being evaluated.

## Conclusions

Both 8-azaguanine and 5350150 impaired HIV-1 expression by altering viral RNA processing through effects at the level of RNA splicing and export. Together, our findings establish that HIV-1 RNA processing can be modulated with small molecules to suppress HIV replication, establishing a new avenue for the treatment of this infection.

